# Tricuspid valve infective endocarditis complicated with multiple lung abscesses and thoracic empyema as different pathogens: a case report

**DOI:** 10.1186/s13019-019-0867-1

**Published:** 2019-02-26

**Authors:** Ya-Fu Cheng, Yung-Kun Hsieh, Bing-Yen Wang, Ching-Yugn Cheng, Chang-Lun Huang

**Affiliations:** 1 Division of general thoracic surgery, department of Surgery, Changhua Christian Hospital, No. 135 Nanxiao St., Changhua City, Changhua County, 500 Taiwan; 2 Division of cardiovascular surgery, department of surgery, Changhua Christian Hospital, Changhua, Taiwan

**Keywords:** Tricuspid valve replacement, Tricuspid valve infective endocarditis, Lung abscesses, Empyema

## Abstract

**Background:**

Only 4.1% of tricuspid valve IE cases require surgical intervention. The complication after tricuspid valve IE with lung abscess and empyema is rare.

**Case presentation:**

We report the case of a 38-year-old male (an intravenous drug abuser) diagnosed with tricuspid valve IE who underwent tricuspid valve replacement. The case was complicated by multiple lung abscesses and thoracic empyema. The pathogens causing the lung abscesses and empyema were *Acinetobacter baumannii complex* and *Candida albicans*, which were different from those causing the endocarditis.

After 4 weeks of antibiotic treatment, chest X-ray revealed bilateral clear lung markings with only mild blunting of the right costophrenic angle.

**Conclusion:**

The pathogen causing the lung abscess is not always compatible with that causing the endocarditis. Thoracoscopic incision of the abscess with 4 to 6 weeks of broad-spectrum antibiotic treatment is effective and safe.

## Background

Only 5–10% of infective endocarditis (IE) cases are reported to be right-sided IE with more than 90% of these cases involving the tricuspid valve [1]. Only 4.1% of total tricuspid valve IE cases require surgical intervention, primarily due to large vegetation, recurrent septic pulmonary emboli, and failure of medical therapy [2]. Most tricuspid valve IE cases are strongly associated with intravenous drug abuse (approximately 30–40% patients have a history of intravenous drug use) [3]. Among these patients who underwent surgical intervention, 30-day mortality and 5-year survival rates were reported to be 12.5 and 63.9%, respectively [4]. Cardiac, neurologic, renal, and musculoskeletal complications are the major complications associated with tricuspid valve IE. Pulmonary complications are rare and may include bacterial pneumonia, lung abscess, pleural effusion, and pneumothorax.

Here, we report the case of a 38-year-old male diagnosed with tricuspid valve IE caused by methicillin-susceptible *Staphylococcus aureus*. The patient underwent tricuspid valve replacement and had a history of intravenous drug abuse. The case was complicated by multiple lung abscesses and thoracic empyema due to carbapenem-susceptible *Acinetobacter baumannii complex* and *Candida albicans* on postoperative day 14, requiring thoracoscopic decortication and incision of the lung abscess.

## Case presentation

A 38-year-old male presented to our emergency room with a 4-day history of intermittent fever and chills without nausea or vomiting. The patient had a past history of intravenous heroin abuse and atrioventricular reentry tachycardia status post radiofrequency catheter ablation. Shortness of breath, cough with some yellowish sputum, tachycardia, low blood pressure (80/40 mmHg under Levophed use), and anuria were noted. The patient had not experienced nausea or vomiting.

**Fig. 1:**
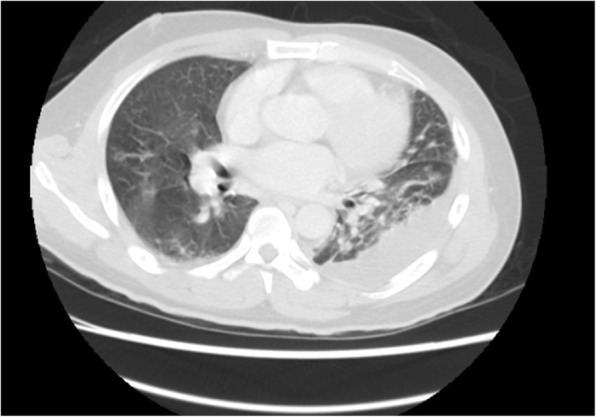
Chest CT shows loculated pleural effusion, consolidations with central lucency collection in both lungs, and mild pericardial effusion

Physical examination revealed bilateral coarse breath sounds and a 4/6 pan systolic heart murmur over the left fourth rib. Laboratory analysis revealed a white blood cell count of 35,030 μL (range: 3500–9100 μL; neutrophilia, 73.4%) and a creatinine level of 3.19 mg/dL (range: 0.70–1.30 mg/dL). Chest X-ray revealed interstitial infiltration with mottled consolidation superimposed on bilateral lung fields and blunting of the left costophrenic angle. Chest computerized tomography (CT) showed loculated pleural effusion, consolidations with central lucency collection in both lungs, and mild pericardial effusion (Fig. 1). Echocardiography revealed normal left ventricle wall motion (left ventricular ejection fraction, 58%) and a floating vegetation in the tricuspid valve with moderate to severe tricuspid regurgitation.

**Fig. 2:**
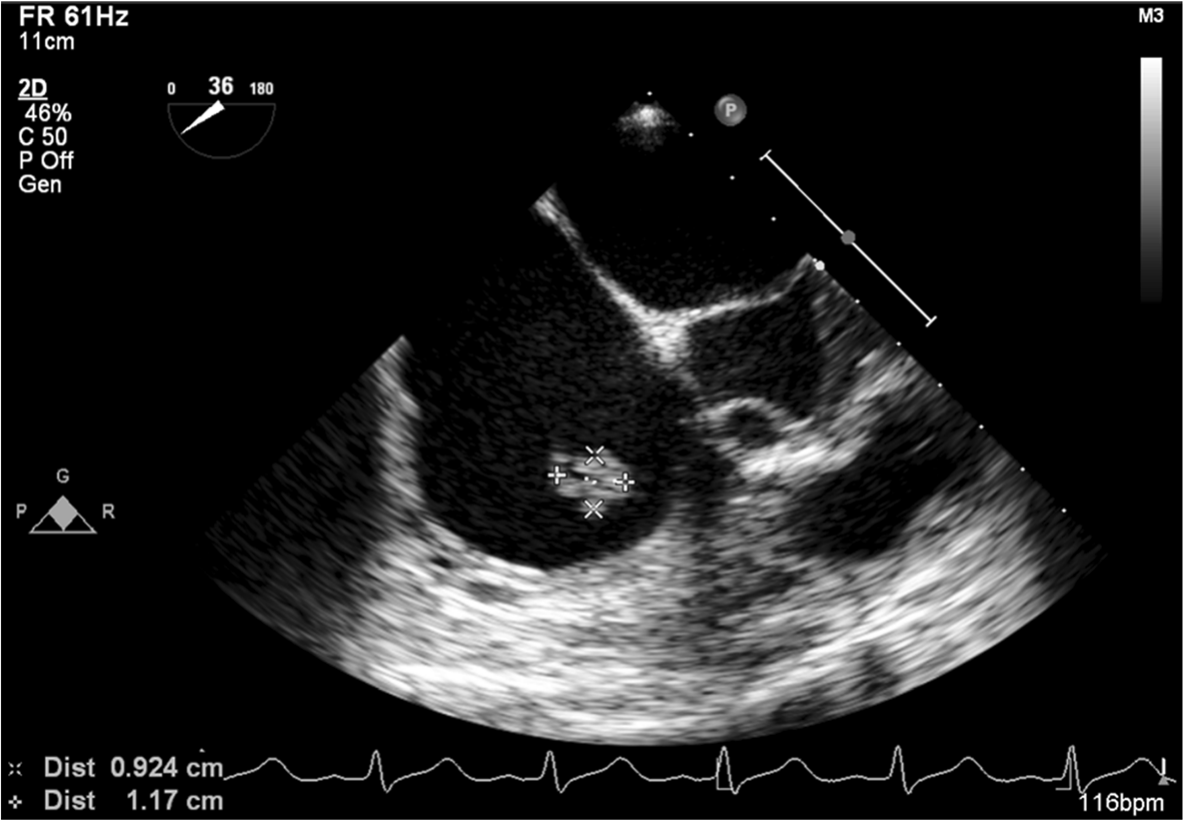
The pre-operative transesophageal echocardiography (TEE) showed 0.9 × 1.2 cm^2^ vegetation over tricuspid valve

Because left empyema and tricuspid valve IE with septic or cardiogenic shock were suspected, left chest tube were inserted and left pleura effusion culture showed methicillin-susceptible *S. aureus*. Right heart failure secondary to severe TR and poor response to medical therapy were noted 1 day after chest tube insertion. An endotracheal tube was insert ion and the patient underwent tricuspid valve replacement with a 33 mm Hancock II tissue valve via median sternotomy with another left chest tube insertion due to all of the anterior chordae tendineae were rupture. The pre-operative transesophageal echocardiography (TEE) showed 0.9 × 1.2 cm^2^ vegetation over tricuspid valve. (Fig. 2).

Blood and sputum cultures showed methicillin-susceptible *S. aureus*. The patient received postoperative antibiotic treatment (oxacillin, 2000 mg six times daily). The early postoperative treatment course was uneventful, with white blood cell count decreased to 14,300 μL and creatinine level decreased to 1.08 mg/dL.

**Fig. 3:**
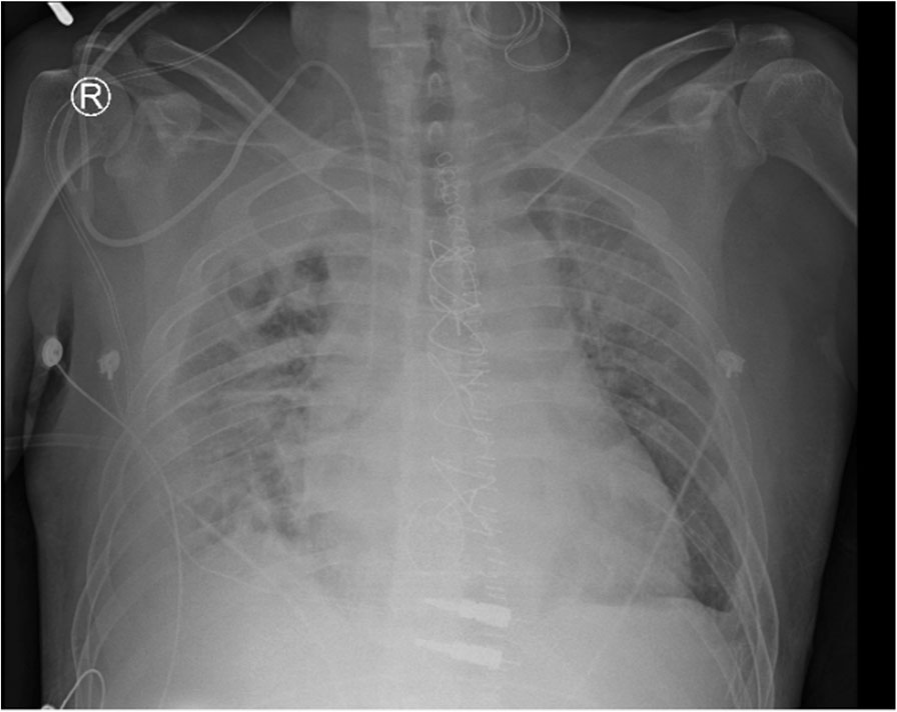
Follow-up chest X-ray 14 days after tricuspid valve replacement shows loculated bilateral pleural effusions with perihilar and lower lung haziness infiltrates

**Fig. 4:**
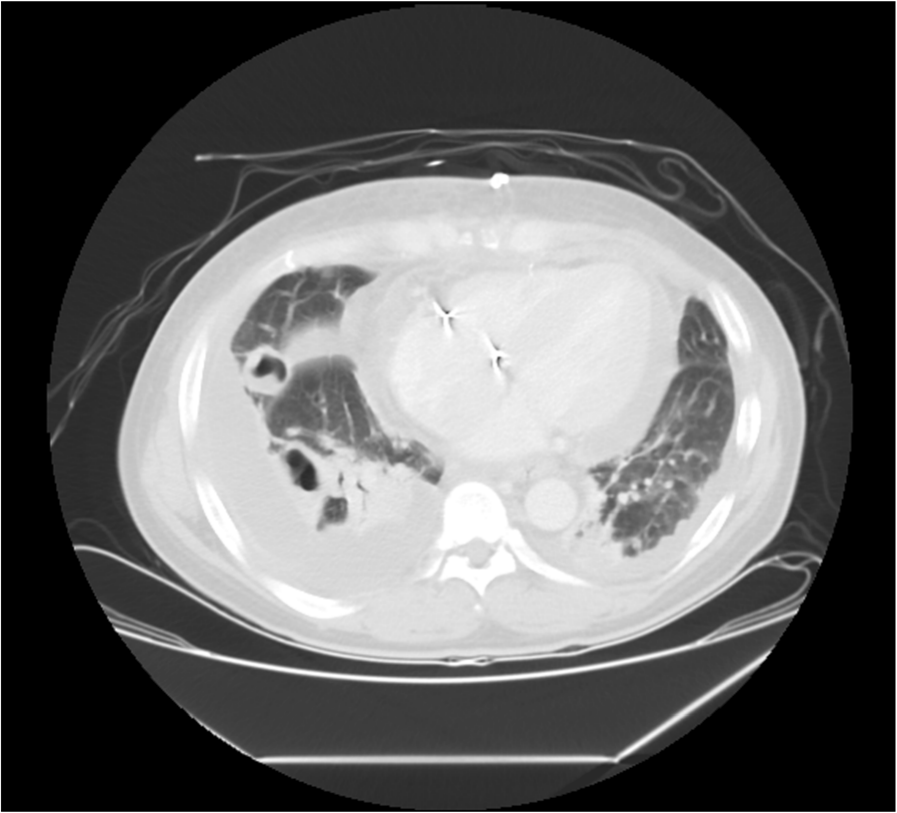
Chest CT reveals multiple cavitary nodules in the bilateral lungs

However, fever up to 38.4 °C and dyspnea were noted on postoperative day 14. Follow up chest X-ray revealed loculated bilateral pleural effusions with perihilar and lower lung haziness infiltrates (Fig. 3). Chest CT revealed multiple cavitary nodules in the bilateral lungs (Fig. 4). The patient underwent thoracoscopic decortication of the right pleura and incision of the right lower lung abscess with postoperative chest tube drainage. Intraoperative pleural fluid and lung abscess cultures showed carbapenem-susceptible *A. baumannii complex* and *C. albicans*. We replaced oxacillin with meropenem 500 mg four times daily and fluconazole 200 mg one time daily. No further fever was noted. After 4 weeks of antibiotic treatment, chest X-ray revealed bilateral clear lung markings with only mild blunting of the right costophrenic angle.

## Discussion and conclusions

The three indication of surgical procedure recommended at 2015 ESC Guidelines were as follow: I) TV vegetations > 20 mm and recurrent septic pulmonary emboli with or without concomitant right heart failure; (II) IE caused by microorganisms that are difficult to eradicate or bacteremia for at least 7 days despite adequate antimicrobial therapy; and (III) Right heart failure secondary to severe TR with poor response to diuretic therapy [1]. Although the lesion is less than 20 mm, this patient is indicated to surgery due to right heart failure secondary to severe TR and poor response to any medical therapy.

There were different surgical approaches of tricuspid valve IE. The European guideline suggested that valve repair is favored than replacement whenever possible without significant destruction [1]. However, in this patient, all of the anterior chordae tendineae were rupture during operation. It’s hard to repair and the patient’s condition could not tolerant one more repair after failure, so tricuspid valve replacement was conducted. Since September 1970, Arbulu et al. reported several right-sided endocarditis with heroin addiction and they received tricuspid valvulectomy without replacement [5]. Although the acceptable outcome was concluded, some patients required further prosthetic heart valve insertion to control medically refractory right-sided heart failure and the others may lead to severe and permanent impairment of right ventricular function. Therefore, the application of valvulectomy without replacement is not that popular in our country.

Tricuspid valve IE complicated by multiple lung abscesses is rare. Yoshimoto et al. [6] first reported the case of a 21-year-old intravenous drug addicted male with right-sided IE complicated by bilateral lung multiple consolidations. However, the culture results were not reported.

Demin et al. [7] further reviewed 100 intravenous drug users with IE, of whom 65 had positive blood cultures. Among these, 46 were positive for *S. aureus* and only one was positive for *A. baumannii complex.* In this study, four cases were complicated by lung abscesses, but no abscess culture was obtained and the blood culture results of these four patients were unclear.

In Taiwan, Liu et al. [8] treated an 86-year-old right-sided IE patient with daptomycin. After 5 days of antibiotic treatment, multiple right lower lung abscesses were found. Sputum and blood cultures were negative. The lung abscesses resolved on chest X-ray after linezolid treatment for 10 days. Chen et al. [9] reported the case of a 21-year-old male with tricuspid valve IE and bilateral multiple lung abscesses, with a blood culture positive for methicillin-susceptible *S. aureus.*

Although some previous studies have reported IE complicated by lung abscesses, no abscess pathogen was reported. Most of these patients had a negative blood culture due to antibiotic treatment for a few days. We believe that these patients were not merely infected by *S. aureus* translocation. Ercan et al. [10] reported the case of a 24-year-old postpartum female with methicillin-resistant *S. aureus* tricuspid valve IE who underwent tricuspid valve replacement. Postoperative chest CT revealed bilateral multiple lung abscesses and repeated blood cultures yielded *Pseudomonas aeruginosa*.

To the best of our knowledge, ours is the first study in which lung abscess cultures were obtained results showed *A. baumannii complex* and *C. albicans.* We assumed that most of these lung abscesses were not infected by the previous IE pathogen. The pathogenesis is unclear. It is possible that this patients’ abscesses were acquired from ventilator-associated pneumonia (VAP) and make sense of these pathogens. However, the frequency of combined *A. baumannii complex* and fungi is not that common. The use of oxacillin or vancomycin is mostly ineffective to cover those pathogens. The antibiotic covered Gram-negative bacteria and thoracoscopic decortication with incision lung abscess is required. Antibiotic coverage for 4 to 6 weeks is adequate for these lung abscess patients.

This report concluded that tricuspid valve IE complicated by multiple lung abscesses and empyema is rare. The pathogen causing the lung abscess is not always compatible with that causing the endocarditis. Thoracoscopic incision of the abscess with 4 to 6 weeks of broad-spectrum antibiotic treatment is effective and safe.

## Abbreviations

CT: computerized tomography
IE: infective endocarditis
